# Proteasome impairment by α-synuclein

**DOI:** 10.1371/journal.pone.0184040

**Published:** 2017-09-25

**Authors:** Lisa Zondler, Marcus Kostka, Patrick Garidel, Udo Heinzelmann, Bastian Hengerer, Benjamin Mayer, Jochen H. Weishaupt, Frank Gillardon, Karin M. Danzer

**Affiliations:** 1 Neurology Department, Ulm University, Ulm, Germany; 2 Boehringer Ingelheim Pharma GmbH Co.KG, Biberach an der Riß, Germany; 3 Institute for Epidemiology and Medical Biometry, Ulm University, Ulm, Germany; Louisiana State University Health Sciences Center, UNITED STATES

## Abstract

Parkinson’s disease (PD) is the second most prevalent neurodegenerative disorder worldwide and characterized by the loss of dopaminergic neurons in the patients’ midbrains. Both the presence of the protein α-synuclein in intracellular protein aggregates in surviving neurons and the genetic linking of the α-synuclein encoding gene point towards a major role of α-synuclein in PD etiology. The exact pathogenic mechanisms of PD development are not entirely described to date, neither is the specific role of α-synuclein in this context. Previous studies indicate that one aspect of α-synuclein-related cellular toxicity might be direct proteasome impairment. The 20/26S proteasomal machinery is an important instrument of intracellular protein degradation. Thus, direct proteasome impairment by α-synuclein might explain or at least contribute to the formation of intracellular protein aggregates. Therefore this study investigates direct proteasomal impairment by α-synuclein both *in vitro* using recombinant α-synuclein and isolated proteasomes as well as in living cells. Our experiments demonstrate that the impairment of proteasome activity by α-synuclein is highly dependent upon the cellular background and origin. We show that recombinant α-synuclein oligomers and fibrils scarcely affect 20S proteasome function *in vitro*, neither does transient α-synuclein expression in U2OS ps 2042 (Ubi(G76V)-GFP) cells. However, stable expression of both wild-type and mutant α-synuclein in dopaminergic SH-SY5Y and PC12 cells results in a prominent impairment of the chymotrypsin-like 20S/26S proteasomal protein cleavage. Thus, our results support the idea that α-synuclein in a specific cellular environment, potentially present in dopaminergic cells, cannot be processed by the proteasome and thus contributes to a selective vulnerability of dopaminergic cells to α-synuclein pathology.

## Introduction

Parkinson’s disease (PD) is a devastating neurodegenerative disease and the most frequent movement disorder in the modern Western society. PD is characterized by loss of dopamine secreting neurons of the substantia nigra pars compacta in the midbrain [1]. So far, the knowledge about the underlying intracellular mechanisms of neuronal toxicity in PD is incomplete. However, the discovery that specific monogenic mutations, e.g. mutations in the *SNCA* gene (encoding α-synuclein), cause genetic forms of PD has contributed markedly to the understanding of the molecular mechanisms of PD pathogenicity [1–3].

Large protein aggregates, the so-called Lewy bodies (LBs) in the patients’ brains are the primary histopathological hallmark of PD as well as of dementia with Lewy bodies (DLB) [4]. The identification of misfolded α-synuclein (α-syn) as the main protein component of LBs has moved α-syn into the focus of LB-disease related research [5, 6]. α-syn is a small acidic protein lacking a defined secondary structure. In the context of neurodegeneration the range of α-syn assemblies comprises monomers, oligomers and protofibrils as well as fibrils and large LB-associated aggregates [7–12]. The physiological functions of α-syn remain elusive to date, however, α-syn has been associated with synaptic function/plasticity, cell differentiation and vesicular trafficking [13]. α-syn dysfunction/overexpression has been linked to impaired intracellular trafficking of endoplasmatic reticulum and golgi vesicles [14–16], altered membrane permeability [17, 18], mitochondrial dysfunction and increased production of reactive oxygen species [19, 20]. Further, impairment of proteasomal activity by aggregated α-syn has been suggested to contribute to α-syn mediated (neuro-) toxicity [21, 22].

The ubiquitin proteasome system (UPS) is a selectively proteolytic system with major impact on cellular protein homeostasis. Classical proteasomes consist of a 20S core that comprises several catalytic subunits, and two 19S caps that mediate binding of ubiquitinated proteins that have been targeted for proteasomal degradation and initiate protein cleavage [22, 23]. Soluble un-/misfolded proteins are targeted to the UPS by polyubiquitination, unfolded into nascent polypeptide chains and while passing through the proteasomal pore the ubiquitinated polypeptide chains are cleaved into short peptides [23]. Thus, proteasomal impairment promotes the formation of intracellular protein aggregates.

In the context of PD, previous studies have suggested that α-syn related toxicity includes direct impairment of proteasomal activity [21, 22]. To test this hypothesis, the aim of the present study was to investigate the effect of different preparations of recombinant α-syn on proteasomal activity both in cell-free systems and in living cells.

## Materials and methods

All chemicals used were purchased from Sigma Aldrich, Inc., Munich, Germany unless stated otherwise.

### Expression and purification of recombinant wild-type α-syn

Expression and purification was performed as Nuscher et al. previously described [24]. Amersham Biosciences, Munich, Germany) and eluted with a 25 mM to 500 mM NaCl salt gradient. T. Briefly, pET-5aα/β-syn wt plasmid was used to transform Escherichia coli BL21(DE3) pLysS (Novagen, Madison, WI, USA). Expression was induced with isopropyl-ß-D-thiogalactopyranose (Promega, Mannheim, Germany) for 4 h. Cells were harvested, resuspended in 20 mM Tris and 25 mM NaCl, pH 8.0 and lysed by freezing in liquid nitrogen followed by thawing. After 30 min of boiling, the lysate was centrifuged at 17600 g for 15 min at 4°C. The supernatant was filtered through 0.22 μm filter (Millex-GV, Millipore, Bedford, MA, USA), loaded onto a HiTrap Q HP column (5 ml, he pooled α-syn peak passed over a Superdex 200 HR10/30 size exclusion column (Amersham Biosciences, Munich, Germany) using 20 mM Tris, 25 mM NaCl, pH 8.0 as running buffer. The pooled α-syn peak was concentrated using Vivaspin columns MWCO 5kD (Vivascience, Stonehouse, UK) and equilibrated to water. The protein concentration was determined using a BCA protein quantification kit (Pierce, Rockford, IL, USA). Aliquots were lyophilized and stored at -80°C.

### Preparation of α-syn oligomers

#### a) Glabe oligomers

Soluble α-syn oligomers were prepared as described previously [25]. Briefly, 1.0 mg of recombinant α-syn was dissolved in 400 μl HFIP and incubated for 10-20 min at RT. 100 μl of the resulting seedless α-syn solution was added to 900 μl ddH2O in a siliconized Eppendorf tube. After 10-20 min incubation at room temperature, the samples were stirred at 500 rpm using a Teflon coated micro stir bar for 24-48 hr at 22°C.

#### b) Jensen Oligomers

α-syn oligomers were generated as published previously [21].

#### c) Wolozin Oligomers

α-syn oligomers were prepared according to a protocol published by Snyder et al [22].

#### d) SIFT Oligomers

Oligomeric α-syn was prepared as described in detail by Kostka et al [26]. Briefly, lyophilized recombinant protein was dissolved in 50 mM Na2HPO4 buffer (pH 7.0) containing 20% ethanol and 10 μM FeCl3 (J.T. Baker, Griesheim, Germany) to a final concentration of 20 nM in a total assay volume of 20 μl. After shaking for 4h, oligomers were used for AFM analysis or in the proteasome assay.

### Atomic force microscopy (AFM)

Sample preparation for AFM was carried out at RT. Samples were mixed gently to suspend any aggregates; 5–6 μL aliquots were placed on freshly cleaved mica (muscovite, Veeco Europe, Dourdan Cedex, France) and incubated for 80–90 s, after which mica was carefully rinsed twice with 100 μL of filtered, deionized water to remove salt and loosely bound proteins. The mica were dried under dry N_2_-gas. Images were obtained with a MultiModeTM SPM (Veeco, Mannheim, Germany) equipped with an E-Scanner and operating in the TappingMode, using etched silicon NanoProbes (model FESP, Veeco Europe, Dourdan Cedex, France). Data were corrected with regard to the sample tip size (Colton RJ 1997 Procedures in Scanning Probe microscopy). Some typical values were: free oscillation amplitude, 0.8–1.8 V; drive frequency, 65–80 kHz; amplitude setpoint, 300–600 mV and scan rates 0.5–1.4 Hz. The measuring conditions were 39–42% relative humidity and 21–23°C.

### Dot blot assay

Dot Blot assay with recombinant α-syn oligomers was performed as previously described [25]. Briefly, 0.2 – 2 μg α-syn oligomers were applied to a nitrocellulose membrane (pore size 0.22 μm, Whatman Protran, Sanford, ME, USA), blocked with 10 (w/v)% non-fat milkin Tris-buffered saline (TBS) containing 0.01 (v/v) % Tween 20 (TBS-T). The membrane was blocked with 10% (w/v) non-fat dried milk in TBS-T (Sigma-Aldrich, St. Louis, MO, USA) at RT for 1 h. After three washes with TBS-T, the membrane was incubated with anti-oligomer antibody A11 (1:100; Invitrogen, Carlsbad, CA, USA) or Syn-1 antibody (1:1000; BD transduction, Franklin Lakes, NJ, USA) overnight at 4 C with gentle agitation. The membranes were then washed three times for 5 min with TBS-T, incubated with horseradish peroxidase conjugated anti-rabbit IgG or anti mouse IgG (1:2000; Jackson Immuno Research Laboratories, Baltimore, PA, USA) in 5 (w/v) % non-fat dried milk in TBS-T and incubated for 1 h at RT. The blots were washed three times with TBS-T and developed with Pierce ECL chemiluminescence kit from Thermo Scientific (Rockford, IL, USA).

### Western blotting of recombinant α-syn oligomers

800 ng of recombinant oligomers were resolved by electrophoresis on a 4–12% Bis-Tris gradient gel (NuPAGE Novex Bis-Tris Gel, Invitrogen, Carlsbad, CA, USA) according to manufacturer’s instructions using NuPAGE MES buffer. After transfer to nitrocellulose membrane (Protran, Schleicher and Schuell, Whatman GmbH, Dassel, Germany) the blot was blocked for 1 h at RT with blocking buffer (I-block, Tropix, Bedford, MA, USA). The blot was probed with ASY-1 antibody (1:500, kind gift of Poul Henning Jensen, University of Aarhus, Denmark) or monoclonal rabbit anti-GAPDH (1:1000; Turku, Finland) for 1 h at RT. Bands were detected using alkaline phosphatase—conjugated anti-rabbit secondary antibodies (1: 5000; Tropix, Bedford, MA, USA) and imaged with VersaDoc imaging system (Bio Rad laboratories GmbH, Munich, Germany).

### *In vitro* 20 S ubiquitin-independent chymotryptic proteasomal activity assay

Aggregated or monomeric α-syn was incubated at various concentrations with purified 20S proteasomes (human erythrocytes, BioMol, Heidelberg, Germany) for 30 min and then a fluorogenic substrate (Suc- LLVY-AMC, BioMol, Heidelberg, Germany) was added. Ten minutes later, the samples were analysed with a GeminiXS SpectraMax fluorescent spectrophotometer (Amersham Biosciences, Amersham, UK) using an excitation wavelength of 360 nm and an emission wavelength of 460 nm.

### U2OS ps 2042 (Ubi(G76V)-GFP) proteasome assay

Cell line U2OS ps 2042 (Ubi(G76V)-GFP) (Fisher BioImage ApS, Denmark; Boston, August 2005) was grown in Dulbecco’s Modified Eagle Medium (DMEM) (life technologies, Carlsbad, CA, USA) with Glutamax-1 (life technologies Carlsbad, CA, USA) containing 1.0 mg/ml Geneticin (G418), 1% (v/v) Penicillin/Streptomycin and 10% FBS. Cells were seeded into 96-well black clear bottom microtiter plates (BD Biosciences, Heidelberg, Germany) at a density of 5000cells/well and cultured overnight. The proteasome assay monitors ubiquitin/proteasome-dependent proteolysis of a green fluorescent protein (GFP)-based substrate. The GFP substrate consists of a mutated uncleavable ubiquitin moiety [Ubi(G76V)] fused to GFP resulting in constitutive degradation of the protein. U2OS ps 2042 [Ubi(G76V)-GFP] cells were treated with different MG132 concentrations (0.56 μM-5 μM) or DMSO control for 24h cells and were then fixed using 2% formaldehyde in PBS as fixation solution. Accumulation of Ubi(G76V)-EGFP was imaged by fluorescence microscopy.

### U2OS transfection

To study the effect of α-syn on proteasome activity U2OS ps 2042 (Ubi(G76V)-GFP) (Fisher BioImage ApS, Denmark; Boston, August 2005) were transiently transfected using FuGENE^®^6 Transfection Reagent (Promega, Madison, USA) according to manufacturer’s instructions. Briefly, FuGENE^®^6 Transfection Reagent was added to serum-free DMEM and incubated for 10 minutes. Plasmid DNA and FuGENE^®^6 reagent were mixed in 1:3 ratio. pcDNA 3.1neo encoding α-syn[wt], α-syn[A30P] or α-syn[A53T] or pcDNA 3.1 plasmid alone were added in equal amounts and incubated for 30 minutes. Finally, the mixture was added to each dish and incubated for 24 hours.

### Virus transduction

Transduction Virus generation and purification were performed as described in detail in [27]_._ Cells were then fixed for staining and image analysis. We have used AAV serotype 5 encoding either for the enhanced green fluorescent protein (EGFP) or nontagged full-length α-syn(wt) were administered. All viral transgenes were under the control of the cytomegalovirus (CMV) promoter. Fresh medium containing AAV at a concentration of 3000 or 10000 viral genomes per cell was added to U2OS ps 2042 (Ubi(G76V)-GFP) and incubated for 48 h at 37°C in 5 v% CO.

### Immunofluorescence staining

U2OS(Ubi(G76V)GFP or SH-SY5Y cells were fixed using 2% formaldehyde and 1 μM Hoechst 33342^™^ (Molecular Probes, Inc., Eugene, USA) in phosphate-buffered saline (PBS) as fixation solution. After washing, the cells were permeabilized and unspecific binding sites were blocked using 0.05% Saponin and 1% bovine serum albumin in PBS. After washing, the primary antibody rabbit antibody against α-syn (ASY-1, kind gift of Poul Henning Jensen, University of Aarhus, Denmark) was added for 1 h at 37°C, followed by another washing step and incubation with the secondary antibody (polyclonal goat anti-rabbit antibody labelled with Alexa-Fluor 647, Molecular Probes, Inc., Eugene, USA; #A-21246) for 1 h at RT. After a final washing step of 50μl/well remained as residual volume.

### SH-SY5Y stable cell line generation

Stable cell lines were generated as described previously [28]. Mock control cell lines were produced to express the unrelated tet-protein (tet-off pUHD15.1; clonetech, Mountain View, CA, USA). Stable SH-SY5Y human dopaminergic neuroblastoma cells expressing α-syn[wt], α-syn[A30P], α-syn[A53T] or the mock vector were maintained at 37°C in 5 v% CO_2_ in high glucose Dulbecco's modifed Eagle's medium (PAA Laboratories GmbH, Pasching, Austria) supplemented with 15% foetal bovine serum (Invitrogen GmbH, Karlsruhe, Germany) and 4 mM glutamine (Invitrogen GmbH, Karlsruhe, Germany) and 1000 μg/ml G418 (PAA Laboratories GmbH, Pasching, Austria).

### *In vitro* 20/26 S ubiquitin-independent chymotryptic proteasomal activity assay of SH-SY5Y cells

250 μg SH-SY5Y cell lysates, as determined by BCA protein assay (Pierce, Rockford, IL, USA) was incubated in assay buffer (10 mM Tris-HCl, pH 7.8, 0.5 mM dithiothreitol, 5 mM MgCl_2_, and 5 mM ATP) for 30 min at 37°C while shaking at 800 rpm. We then added a fluorogenic substrate (Suc-LLVY-AMC, BioMol, Heidelberg, Germany) and incubated samples for additional 30 min at 37°C while shaking at 800 rpm. Solutions were analyzed using an excitation wavelength of 360 nm and an emission wavelength of 460 nm with the GeminiXS SpectraMax spectrophotometer (Amersham Biosciences, Amersham, UK).

### Transfection of PC12 cells

PC12 cells were obtained from Uwe Knippschild (Ulm University, Ulm, Germany; Ulm, June 2017) and kept in DMEM + 2% FCS. PC12 cells were transfected using Lipofectamine 2000 strictly according to the manufacturer's instructions. Briefly, cells were seeded in 12-well plates and 5 μg of DNA and 5 μl of Lipofectamine 2000 was used per well. Cells were lyzed 20 h post transfection.

### *In vitro* 20S ubiquitin-independent proteasomal activity assay of PC12 cells

The *In Vitro* 20S Ubiquitin-independent Proteasomal Activity Assay of PC12 cells (Millipore, Billerica, Massachusetts, US) was performed strictly according to the manufacturer's instructions. After 20 h of protein expression, PC12 cells were harvested and lyzed in 50mM Hepes + 5mM EDTA + 150 mM NaCl + 1 v% TritonX + 2 mM ATP. The protein content was determined by BCA protein assay (Pierce, Rockford, IL, USA), 45μl of lysate per well was used for the proteasome activity assay and the results were normalized to protein content. The provided positive control and Lactacystin inhibitor were used to control the functionality of the assay. Samples implying the proteasome inhibitor were pre-incubated with Lactacystin for 15 min at RT before the fluorogenic assay substrate was added. After 2 h of incubation at 37°C the assay plate was read in a fluorometer (380/460 filter set). Assays were measure in duplicates, measurements were performed of 4 independent transfections and RFU results were normalized to the respective protein content.

### Western blotting of PC12 lysates

20 μg of cell lysates were resolved by electrophoresis on a 12% Bis-Tris gel (NuPAGE Novex Bis-Tris Gel, Invitrogen, Carlsbad, CA, USA) according to manufacturer’s instructions using NuPAGE MOPS buffer. After transfer to nitrocellulose membrane (Protran, Schleicher and Schuell, Whatman GmbH, Dassel, Germany) the blot was blocked for 1 h at RT with blocking buffer (I-block, Tropix, Bedford, MA, USA). The blot was probed with a mouse anti-synuclein antibody (1:500; BD, Heidelberg, Germany) or anti-rabbit b-actin antibody (1:2000, Thermo, Rockford, IL, USA) for 1h at RT. Bands were detected using HRP—conjugated anti-rabbit/mouse secondary antibodies (1: 5000; Tropix, Bedford, MA, USA) and imaged with VersaDoc imaging system (Bio Rad laboratories GmbH, Munich, Germany).

### Measurement of the assay plates in the IN Cell Analyzer 3000

Automated confocal fluorescence microscopy using the IN Cell Analyzer 3000^™^ (GE Healthcare Bio-Sciences, Little Chalfont, UK) has been described in detail [29]. For our experiments, we employed the 364 laser line combined with a 450BP25 emission filter for Hoechst 33342^™^, the 647 nm laser line combined with a 695BP55 emission filter for Alexa Fluor 647. Fluorescence emission was recorded separately in the blue and red green channels, applying flat field correction for inhomogeneous illumination of the scanned area for each of the three channels.

### Image analysis

Images were analyzed using the nuclear trafficking (TRF2) algorithm of the IN Cell Analyzer 3000^™^. Briefly, the algorithm identified the nuclei as pixel accumulations above a specified intensity threshold in the blue (nuclear) channel image. The number of nuclei corresponds to the absolute cell number. In a specified dilated “cytoplasmic” mask region around these nuclei, the algorithm then searched for cells above a defined threshold in the red channel image, thereby only including successfully transfected cells into further analysis. Cells with Ubi(G76V)GFP signals were identified and the value “%positive cells” for each image was calculated as follows:

$$positive\;cells=\frac{number\;of\;positive\;cells\;in\;signal\;channel}{number\;of\;total\;cells\;in\;signal\;channel}x\text{100}$$

For each treatment the numbers of % positive cells and total cells were normalized to the respective solvent controls to give % of control values [% CTL].

### Statistical analysis

Statistical analysis was performed using Graphpad PRISM software 6. To assess effects of α-syn oligomers on 20S proteasomal activity *in vitro* and in living SH-SY5Y cells, time-dependent group differences were analyzed using a two-way ANOVA approach with repeated measurements.

## Results

### Characterization of different recombinant α-syn oligomer preparations and their effects on 20S proteasomal activity *in vitro*

**Fig 1:**
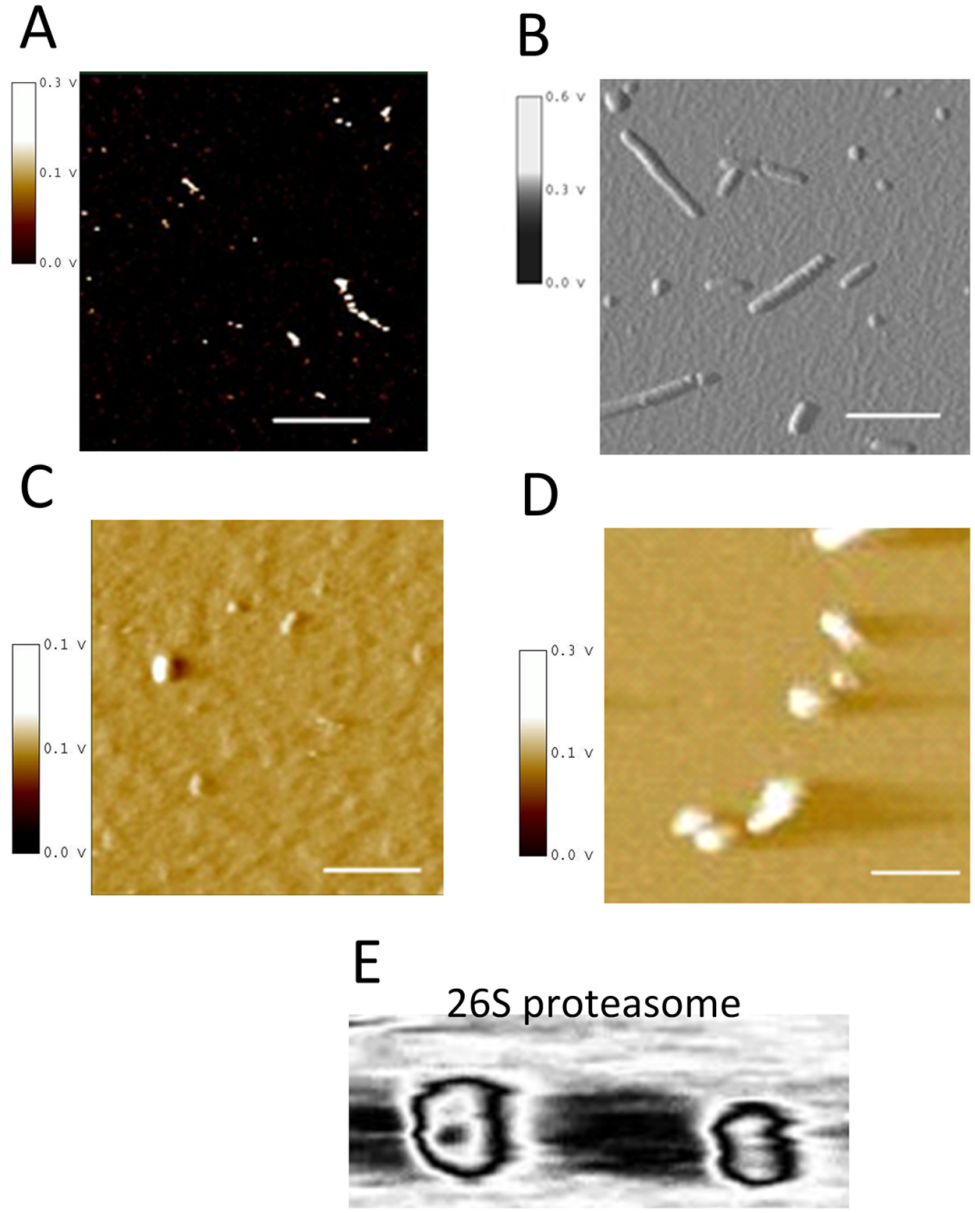
Characterization of different recombinant α-syn oligomer preparations. Preparations of recombinant α-syn were produced according to four different protocols as described previously. AFM analysis of α-syn oligomers produced according to Kayed et al. [25] (A), Lindersson et al. [21] (B), Snyder et al. [22] (C) and Kostka et al. [26] (D) reveals that all preparations display different forms of oligomers and protofibrils. Presented are images (500 nm x 500 nm) in AFM amplitude mode, scale bars = 125 nm. (E) AFM analysis of 20S proteasomes isolated from human erythrocytes demonstrates no structural modifications by α-syn oligomers generated according to Kostka et al.

To assess the effect of recombinant α-syn on proteasomal activity we produced α-syn assemblies by four independent methods and characterized all α-syn preparations using immunoblotting and atomic force microscopy (AFM) (Fig 1A–1D, S1A and S1B Fig. Characterization of recombinant α-syn preparations). AFM demonstrates that all generated α-syn preparations comprised a mixture of different oligomeric and fibrillar species and varied regarding α-syn oligomer composition (Fig 1A–1D). To confirm that 20S proteasomes were successfully isolated from human erythrocytes, morphologically intact proteasomes were depicted by AFM (Fig 1E).

**Fig 2:**
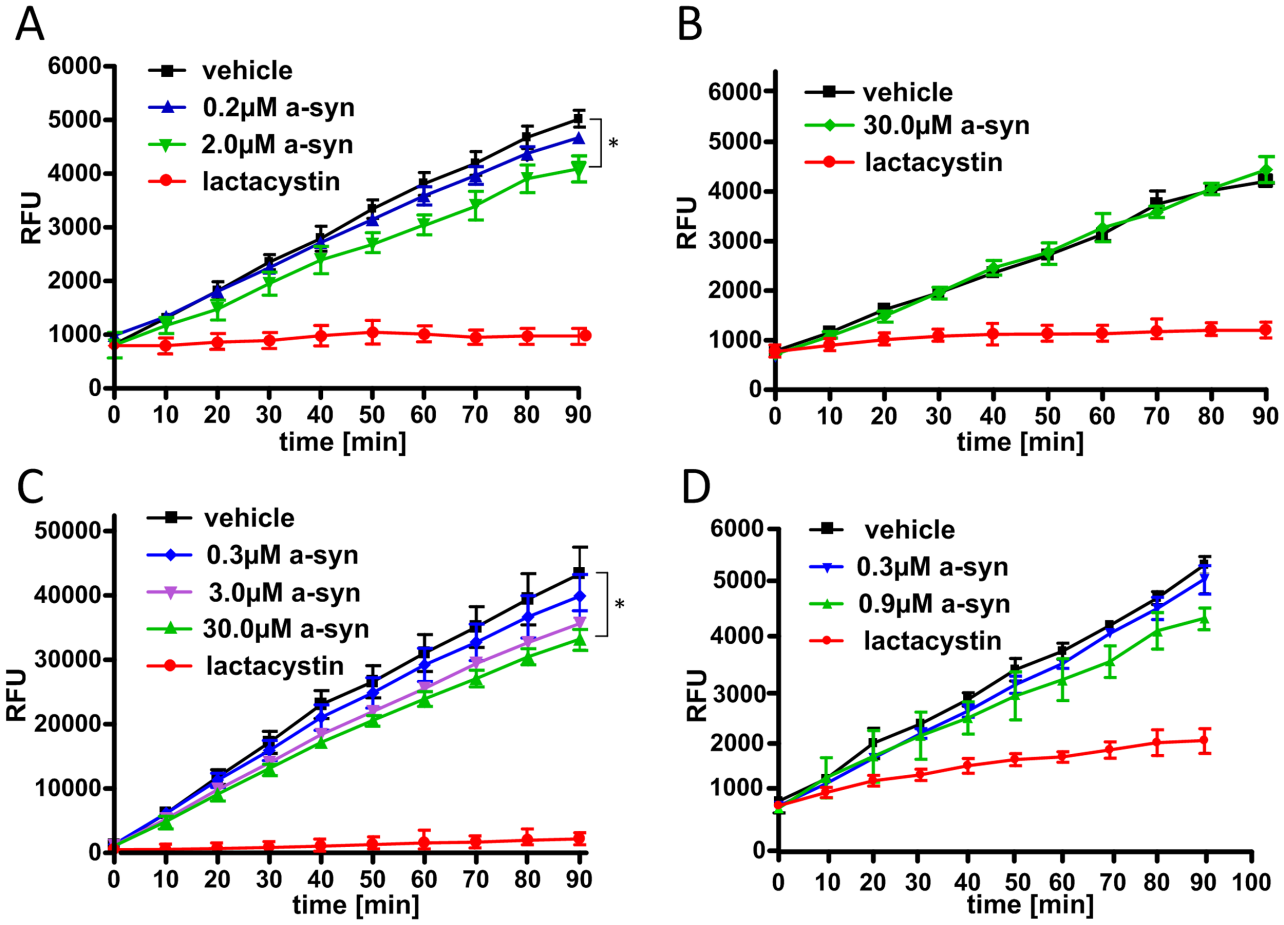
Effects of recombinant α-syn oligomers on 20S proteasomal activity *in vitro*. To assess the effect of recombinant α-syn on 20S proteasomal activity a fluorogenic cell-free 20S proteasome assay was performed, implementing different α-syn preparations at various concentrations as well as a negative vehicle control and a positive lactacystin control. No or little inhibitory effect on the catalytic activity of isolated human 20S proteasomes was observed for all four α-syn oligomer preparations: (A) Kayed et al. [25] (0.2 μM α-syn vs vehicle, p = 0.6008; 2.0 μM α-syn vs vehicle, p = 0.0405) (B) Lindersson et al. [21] (30 μM α-syn vs vehicle, p = 0.8088) (C) Snyder et al. [22] (0.3 μM α-syn vs vehicle, p = 0.4838; 3.0 μM α-syn vs vehicle, p = 0.0735; 30.0 μM α-syn vs vehicle, p = 0.0194) (D) Kostka et al. [26] (0.3 μM α-syn vs vehicle, p = 0.3167; 0.9 μM α-syn vs vehicle, p = 0.0603). All graphs display mean comparable relative fluorescence intensities (RFU) +/- SD, n = 3).

To measure the effect of the different α-syn preparations on 20S proteasomal activity the cell-free fluorogenic 20S proteasome assay was performed. Here, the ubiquitin-independent chymotrypsin-like proteolytic activity was analyzed over time upon application of the four α-syn preparations in different concentrations. Lactacystin, a potent inhibitor of the chymotrypsin-like proteasomal activity was implemented as a positive control (Fig 2). Time-dependent group differences were analyzed using a two-way ANOVA approach with repeated measurements. Using this approach only limited effects of recombinant α-syn on 20S proteasome activity was observed for the four α-syn preparations compared to the respective vehicle controls (Fig 2A–2D).

### Effects of α-syn overexpression on 26S proteasomal activity in living cells

**Fig 3:**
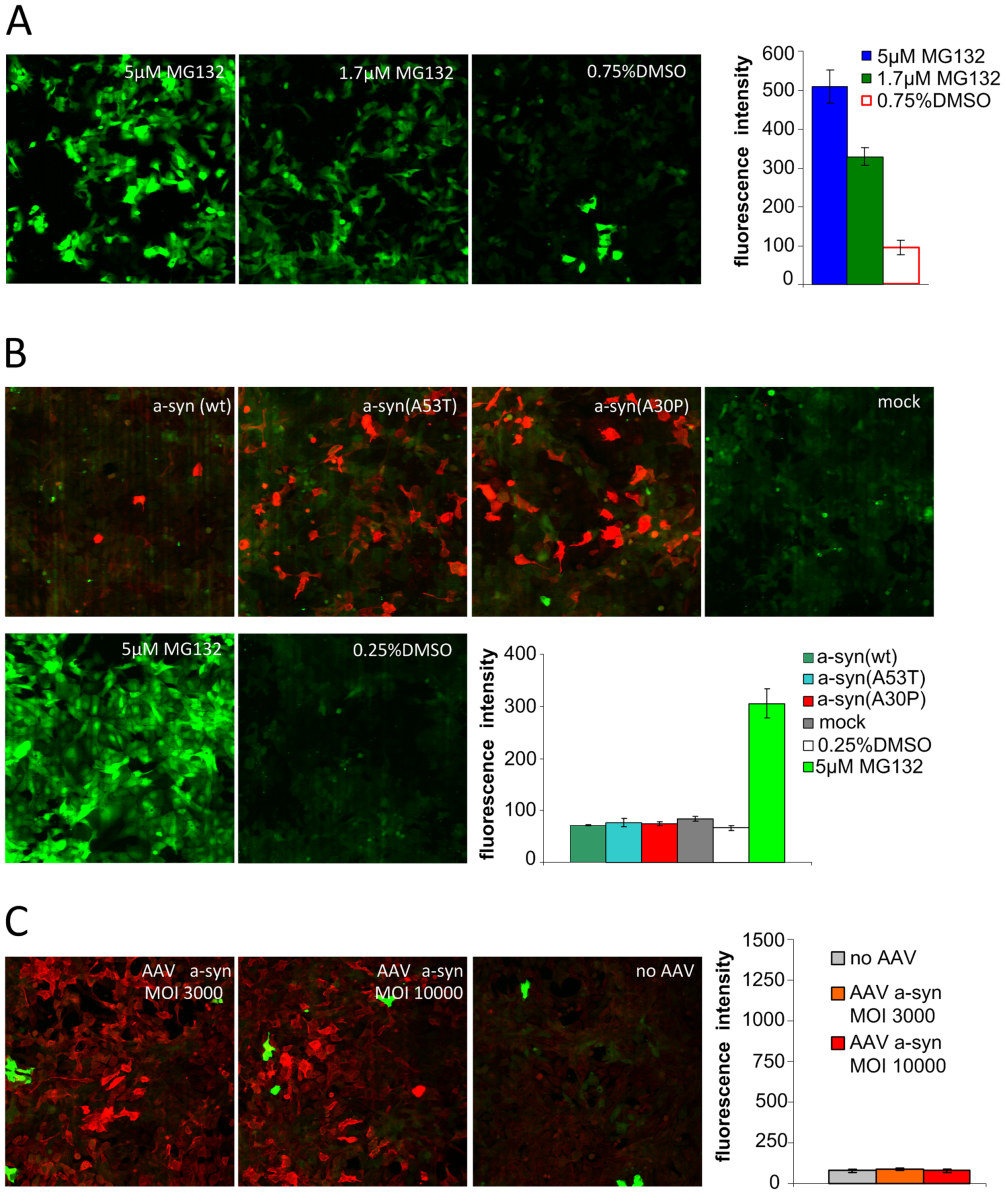
Effects of α-syn expression on 26S proteasomal activity in living cells. A cellular Ubi(G76V)-GFP proteasome assay was performed to measure the effect of α-syn on proteasomal activity in living cells transfected with constructs transiently expressing α-syn. (A) The validity of the system was confirmed by application of MG132, a membrane permeable inhibitor of proteasomal activity as indicated by dose dependent intensity of green fluorescence. (B) Representative images showing U2OS ps 2042 (Ubi(G76V)-GFP) cells transfected with α-syn(wt), α-syn(A30P) or α-syn(A53T), respectively, as well as the mock and vehicle controls. α-syn expression is indicated by red fluorescence. Quantification of green fluorescence as a measure of proteasomal impairment reveals no effect of transfection with α-syn encoding plasmids on proteasomal activity in this assay. (C) Representative images showing U2OS ps 2042 (Ubi(G76V)-GFP) cells infected with α-syn expressing AAVs. Concordantly, quantification of green fluorescence as a measure of proteasomal impairment reveals no effect of viral α-syn expression on proteasomal activity in this Ubi(G76V)-GFP proteasome assay.

To assess whether α-syn overexpression had an effect on proteasomal activity in living cells we performed a cellular Ubi(G76V)-GFP proteasome assay. U2OS ps 2042 (Ubi(G76V)-GFP) cells express G76V mutated ubiquitin tagged to GFP. In case of proteasomal activity, the GFP tags are constantly degraded and fluorescence is impeded, therefore measurement of green fluorescence can be used to quantify intracellular proteasomal activity. Application of MG132, a membrane permeable inhibitor of proteasomal activity confirmed the validity of the system (Fig 3A).

To assess the effect of α-syn on intracellular proteasomal activity U2OS ps 2042 (Ubi(G76V)-GFP) cells were transfected with plasmids encoding wild-type α-syn as well as α-syn harboring PD causative mutations [α-syn (wt), α-syn(A53T), α-syn(A30P)]. To ensure α-syn expression immunofluorescence staining using a primary anti-α-syn antibody (ASY-1) and secondary antibodies emitting red fluorescence was performed. To quantify the proteasome activity, green fluorescence was quantified in transfected cells (identified by RFP fluorescence) using an automated confocal microscopy platform. MG132 and vehicle treated cells as well as cells transfected with a mock plasmid were used as controls (Fig 3B). Using this approach, we found no effect of α-syn (wt) nor α-syn(A53T) or α-syn(A30P) expression on the proteasomal activity in U2OS ps 2042 (Ubi(G76V)-GFP) cells.

Next, we further affirmed the finding that expression of α-syn does not impair proteasomal activity in this model by transducing U2OS ps 2042 (Ubi(G76V)-GFP) cells using α-syn (wt) expressing AAV vectors (Fig 3C).

### Effects of stable α-syn expression on 20/26S proteasomal activity in SH-SY5Y cells

**Fig 4:**
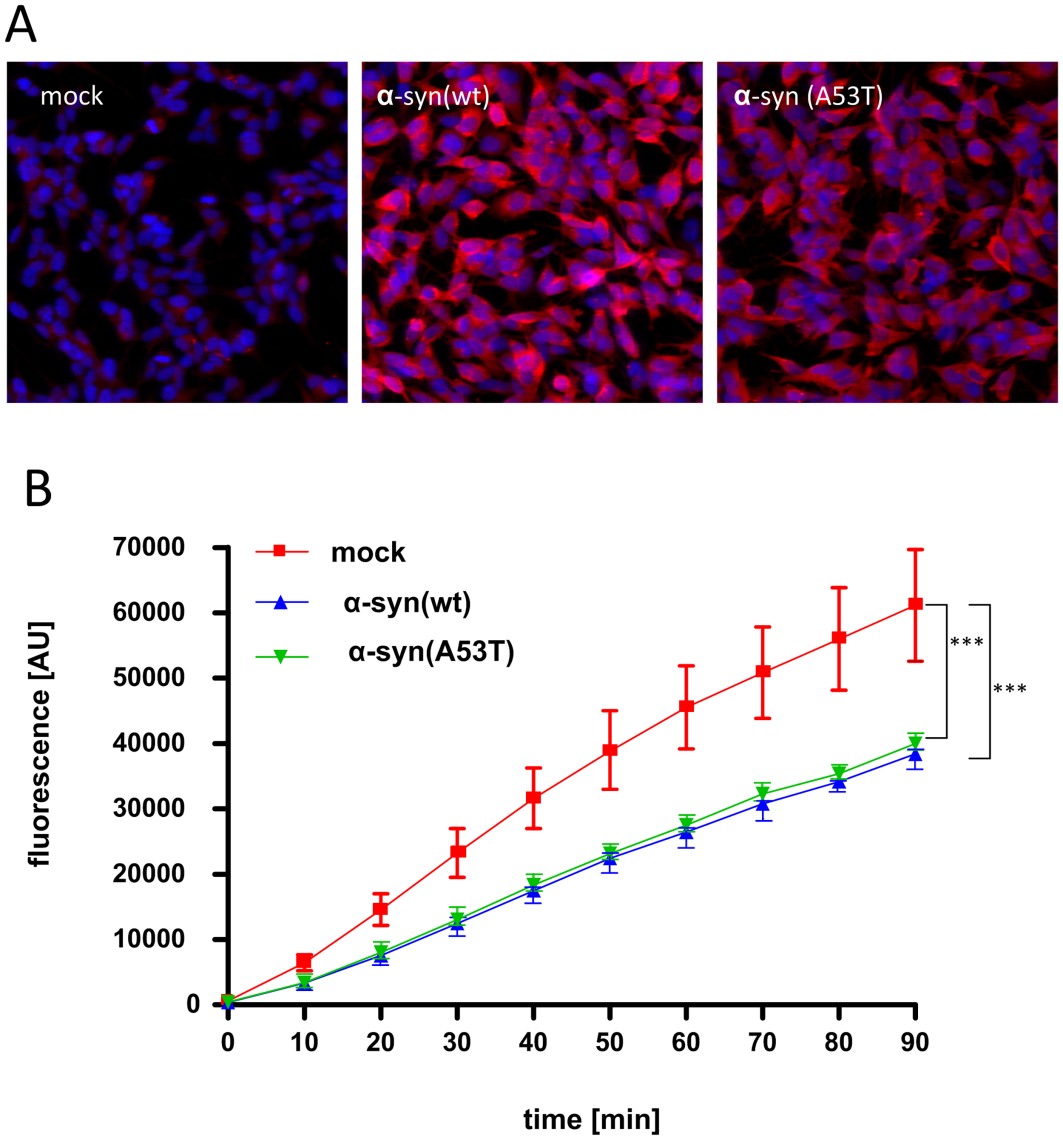
Effects of α-syn expression on 20/26S proteasomal activity in living SH-SY5Y cells. A cellular 20/26S proteasome assay implementing SH-SY5Y cells that stably express wild-type or A53T α-syn as well as a mock control was performed to assess the effect of the presence of α-syn on proteasomal activity on a neuronal background. (A) Immunocytochemistry confirms comparable levels of α-syn expression in wild-type- and A53T α-syn expressing SH-SY5Y cells indicated by red fluorescence. (B) Both expression of wild-type and A53T α-syn results in decreased proteasomal activity as indicated by decreased relative fluorescence intensity (RFU) in this setting. Time-dependent group differences were analyzed using a two-way ANOVA approach with repeated measurements, wild-type α-syn vs control, p<0.001 and α-syn A53T vs control, p<0.001, depicted are the mean +/- SD, n = 3.

PD primarily affects dopaminergic neurons in the substantia nigra pars compacta of the midbrain. Therefore we also tested the effect of α-syn on proteasome activity in SH-SY5Y cells, a dopaminergic neuroblastoma cell line. To that end we generated SH-SY5Y cell lines stably expressing wild-type and A53T α-syn as well as a mock control cell line expressing the unrelated tet transactivator protein and performed a fluorogenic 20/26S proteasome assay. Interestingly, in this assay, expression of both wild-type and A53T α-syn resulted in a time-dependent and highly significant decrease of the chymotrypsin-like 20S/26S UPS activity (Fig 4A, p<0.001). Immunoblotting was performed ensuring that cellular proteasomes in the α-syn expressing SH-SY5Y cells was not decreased (data not shown).

Immunocytochemistry confirmed similar expression of wild-type and A53T α-syn (Fig 4A). Importantly, similar inhibitory effects of proteasomal impairment by α-syn (wild-type and A30P) were observed in transiently transfected PC12 cells (S2A and S2B Fig. Effect of α-syn expression on 20/26S proteasomal activity in living PC12 cells).

## Discussion

Aside from other cellular and molecular aspects of α-syn related neuronal toxicity, both direct and indirect impairment of the UPS by α-syn have been suggested to contribute to neurodegeneration in PD [21, 22, 30–34]. However, α-syn related inhibition of UPS functionality turns out not to be linear and—due to partially controversial results—to be dependent upon the applied α-syn species, α-syn dose and mode of application as well as the applied proteasome assay [21, 22, 30, 34]. However, taken together our and previously published work suggests an interrelation of α-syn abundance and UPS dysfunction to be one important aspect of α-syn mediated toxicity, particularly in dopaminergic neurons.

The first hint of UPS impairment in α-syn related neurodegeneration including PD, MSA and DLB came from the observation that the occuring LBs are strongly positive for ubiquitin [21]. Further, Lindersson et al described colocalization of proteasomal subunits and LBs in midbrain neurons of PD and DLB patients and even direct binding of α-syn filaments (not monomers) to the proteasomal 20S subunit [21]. Snyder et al additionally showed that both monomeric and aggregated α-syn bind to the proteasomal 19S subunit [22] *in vitro*. Investigating 20S proteasome impairment *in vitro* Snyder et al. describe a dose-dependent impairment by both monomeric and aggregated α-syn [22]. This effect was further specified by Lindersson et al who differentially assessed the activity of the three main enzymatic functions of 20S proteasome upon treatment with α-syn filaments. Their experiments revealed that specifically the chymotrypsin-like protein cleavage activity and at to a lower extent the trypsin-like activity of the 20S proteasome was inhibited by α-syn filaments [21], whereas the caspase-like proteasome function was not affected. Our experiments confirm the results obtained by Snyder et al, however, in our hands, α-syn preparations produced according to Lindersson et al did not impair 20S proteasome activity significantly in a cell-free assay *in vitro*. Together, the four different recombinant α-syn preparations applied to our cell-free assay had no or a rather mild effect on isolated 20S proteasomes *in vitro*. This discrepancy could be explained by the different nature and/or composition of α-syn species and the proteasome isolation method used by different groups (extensively discussed in [21]) and supports the assumption that α-syn might not impair 20S proteasome function in general. Another explanation could be the absence of the 19S subunits in this 20S proteasome assay as α-syn has been shown to specifically bind the 19S cap [22].

In order to put the system in a more physiological context we next sought to test proteasome inhibition by α-syn in living cells. We did not observe an α-syn induced proteasome dysfunction in U2OS ps 2042 (Uibi(G76V)-GFP) cells upon α-syn expression neither by transient transfection nor viral transduction. Assuming that α-syn specifically impairs the chymotrypsin-like proteasome function [21] the effect of α-syn on 20S/26S proteasome function in this U2OS ps 2042 (Ubi(G76V)-GFP) assay could have been masked since only the overall proteasome function can be analyzed in this system. Of note, it is not known which forms of oligomeric α-syn are formed in living cells and according to ours and previous results using preparations of recombinant α-syn, UPS impairment by α-syn is likely to be oligomer species specific. Importantly, using SH-SY5Y cell lines that stably express wild-type or mutant α-syn we observed a strong reduction of the proteasomal chymotrypsin-like protein cleavage function without further stimulation or treatment. Notably, we also observed an impairment of proteasome function in dopaminergic PC12 cells transiently expressing different forms of α-syn. Further, a particular sensitivity of TH-positive neurons to UPS impairment induced by MG132/lactacystin and an increased sensitivity to proteasomal impairment by expression of α-syn has been reported before [30, 35, 36]. In line with our results these studies suggest that α-syn related UPS impairment is not only specific for the chymotrypsin-like proteasome function but also highly dependent upon the cellular context/origin and probably the particular α-syn species. If so, the specific sensitivity to UPS impairment of TH-positive midbrain neurons could significantly contribute to the selective vulnerability of those cells to α-syn-related toxicity in PD. Further studies are needed to resiliently prove a specific predisposition of dopaminergic cells to proteasome impairment by α-syn. UPS impairment has been suggested for other aggregating proteins such as huntingtin, and even generally for β-sheet-like structures [21, 31]. However, in line with our results, the finding that genetic depletion of 26S proteasomes *in vivo* causes neurodegeneration with PD/DLB-like symptoms [37, 38] supports a particular role of UPS dysfunction in α-syn related neurodegeneration.

Therefore, we conclude, that α-syn-related UPS impairment is not a general mechanism, but rather a specific inhibition of the chymotrypsin-like proteasome function that is probably dependent upon the specific composition of α-syn species and the cellular environment. Further, as α-syn-related toxicity specifically affects dopaminergic neurons in the patients’ brains, we assume that direct UPS impairment by α-syn in dopaminergic neurons could make a significant contribution to the selective vulnerability of these cells in PD.

## Supporting information

S1 Fig A and B. Characterization of recombinant α-syn preparations. (PDF)

S2 Fig A and B. Effect of α-syn expression on 20/26S proteasomal activity in living PC12 cells. (PDF)
