# Supplementary material for: Proteasome impairment by α-synuclein
Source: PLoS One. 2017 Sep 25;12(9):e0184040. doi: 10.1371/journal.pone.0184040 (PMC5612461; doi:10.1371/journal.pone.0184040)
Supplement: S1 Fig — (PDF) [file pone.0184040.s001.pdf]

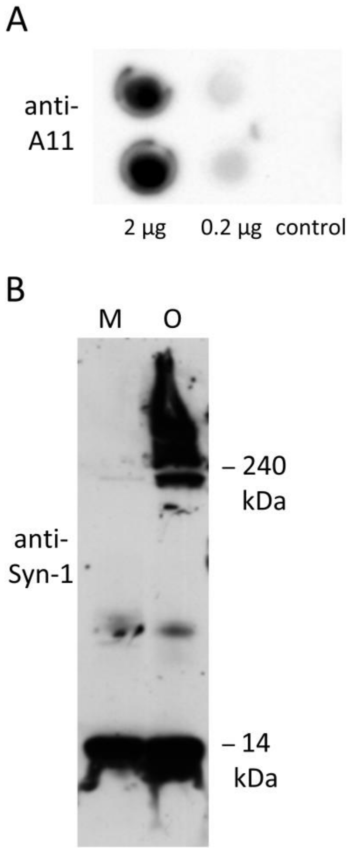

S1 Fig: Characterisation of recombinant α-syn preparations

(A) Dot blot characterisation of α-syn oligomers produced according to Kayed et al. using the oligomer specific antibody A11 confirms the dose-dependence of α-syn oligomers in the preparation. **(B)** The α-syn preparation produced as described by Snyder et al. comprised monomeric and oligomeric forms of the protein as detected by immunoblotting using an α-syn specific antibody.
