# Supplementary material for: Proteasome impairment by α-synuclein
Source: PLoS One. 2017 Sep 25;12(9):e0184040. doi: 10.1371/journal.pone.0184040 (PMC5612461; doi:10.1371/journal.pone.0184040)
Supplement: S2 Fig — (PDF) [file pone.0184040.s002.pdf]

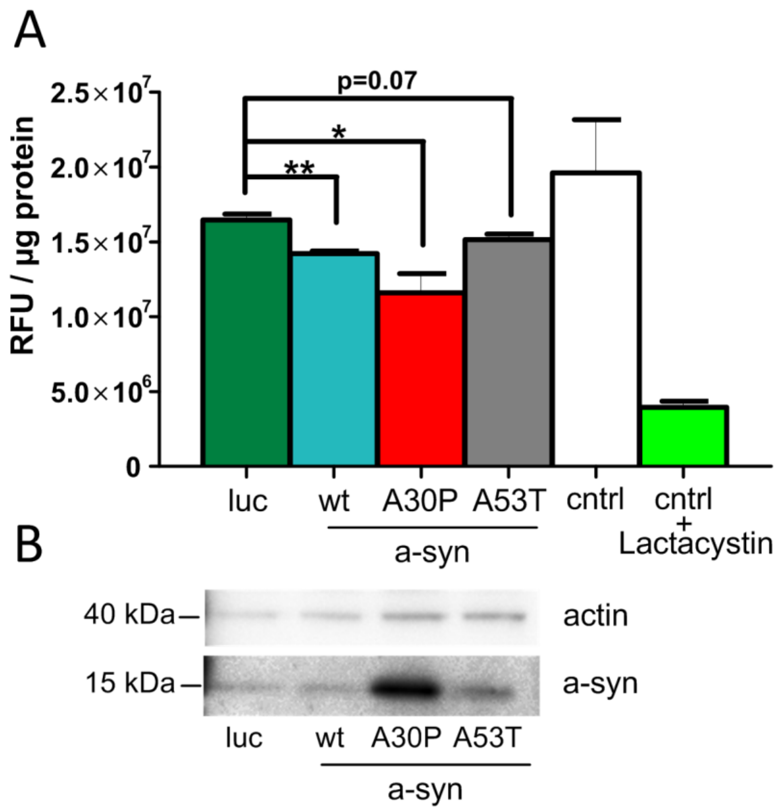

S2 Fig: Effects of α-syn expression on 20/26S proteasomal activity in living PC12 cells

A cellular 20/26S proteasome assay implementing PC12 cells transiently expressing wild-type or A53T α-syn as well as a control protein (Gaussia Luciferase; Luc) was performed to assess the effect of the presence of α-syn on proteasomal activity on a dopaminergic background. **(A)** Both expression of wild-type and A30P α-syn results in decreased proteasomal activity as indicated by decreased relative fluorescence intensity (RFU) per µg protein in this setting. Group differences were analyzed applying unpaired t-tests, wild-type α-syn vs control, p=0.006, α-syn A30P vs control, p=0.02, α-syn A53T vs control, p=0.07, depicted are the mean +/- SD, n=4. **(B)** Immunoblotting of the respective cell lysates using Syn1 and actin antibody.
